# Ethyl 4-(2-hydroxy­ethyl­amino)-3-nitro­benzoate

**DOI:** 10.1107/S1600536810008147

**Published:** 2010-03-17

**Authors:** Aisyah Saad Abdul Rahim, Shafida Abd Hamid, Shivanagere Nagojappa Narendra Babu, Wan-Sin Loh, Hoong-Kun Fun

**Affiliations:** a School of Pharmaceutical Sciences, Universiti Sains Malaysia, 11800 USM, Penang, Malaysia; b Kulliyyah of Science, International Islamic University Malaysia (IIUM), Jalan Istana, Bandar Indera Mahkota, 25200 Kuantan, Pahang, Malaysia; c X-ray Crystallography Unit, School of Physics, Universiti Sains Malaysia, 11800 USM, Penang, Malaysia

## Abstract

In the title compound, C_11_H_14_N_2_O_5_, the mol­ecular structure is stabilized by an intra­molecular N—H⋯O hydrogen bond, which generates an *S*(6) ring motif. The nitro group is twisted slightly from the attached benzene ring, forming a dihedral angle of 5.2 (2)°. In the crystal packing, inter­molecular O—H⋯O and C—H⋯O hydrogen bonds link the mol­ecules into a three-dimensional network. The crystal studied was a non-merohedral twin, the refined ratio of the twin components being 0.264 (2):0.736 (2).

## Related literature

For background to benzimidazoles, see: Mayer *et al.* (1998 ▶); Brouillette *et al.* (1999 ▶); Williams *et al.* (1995 ▶); Wright (1951 ▶). For reference bond-length data, see: Allen *et al.* (1987 ▶). For related structures, see: Narendra Babu, Abdul Rahim, Abd Hamid *et al.* (2009 ▶); Narendra Babu, Abdul Rahim, Osman *et al.* (2009 ▶). For hydrogen-bond motifs, see: Bernstein *et al.* (1995 ▶). For the stability of the temperature controller used for the data collection, see: Cosier & Glazer (1986 ▶).
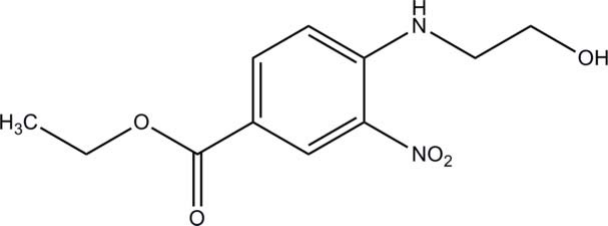


## Experimental

### 

#### Crystal data

C_11_H_14_N_2_O_5_
*M* *_r_* = 254.24
Monoclinic, 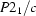

*a* = 10.6422 (6) Å
*b* = 14.9954 (9) Å
*c* = 7.1975 (4) Å
β = 99.607 (2)°
*V* = 1132.50 (11) Å^3^
*Z* = 4
Mo *K*α radiation
μ = 0.12 mm^−1^
*T* = 100 K
0.43 × 0.13 × 0.03 mm

#### Data collection

Bruker SMART APEXII CCD area-detector diffractometer
Absorption correction: multi-scan (*SADABS*; Bruker, 209 ▶0) *T*_min_ = 0.951, *T*_max_ = 0.997
8457 measured reflections
2587 independent reflections
2026 reflections with *I* > 2σ(*I*)
*R*_int_ = 0.050

#### Refinement

*R*[*F* ^2^ > 2σ(*F* ^2^)] = 0.051
*wR*(*F* ^2^) = 0.129
*S* = 1.04
2587 reflections
173 parameters
H atoms treated by a mixture of independent and constrained refinement
Δρ_max_ = 0.50 e Å^−3^
Δρ_min_ = −0.31 e Å^−3^

### 

Data collection: *APEX2* (Bruker, 2009 ▶); cell refinement: *SAINT* (Bruker, 2009 ▶); data reduction: *SAINT*; program(s) used to solve structure: *SHELXTL* (Sheldrick, 2008 ▶); program(s) used to refine structure: *SHELXTL*; molecular graphics: *SHELXTL*; software used to prepare material for publication: *SHELXTL* and *PLATON* (Spek, 2009 ▶).

## Supplementary Material

Crystal structure: contains datablocks global, I. DOI: 10.1107/S1600536810008147/wn2377sup1.cif

Structure factors: contains datablocks I. DOI: 10.1107/S1600536810008147/wn2377Isup2.hkl

Additional supplementary materials:  crystallographic information; 3D view; checkCIF report

## Figures and Tables

**Table 1:** Hydrogen-bond geometry (Å, °)

*D*—H⋯*A*	*D*—H	H⋯*A*	*D*⋯*A*	*D*—H⋯*A*
N2—H2*A*⋯O2	0.84 (3)	1.99 (3)	2.642 (2)	134 (2)
O5—H5*B*⋯O3^i^	0.83 (3)	2.02 (3)	2.851 (2)	177 (3)
C8—H8*A*⋯O5^ii^	0.97	2.51	3.271 (3)	135
C10—H10*A*⋯O5^iii^	0.97	2.54	3.267 (3)	132
C10—H10*B*⋯O1^iv^	0.97	2.43	3.168 (3)	133
C11—H11*A*⋯O2^v^	0.97	2.59	3.403 (3)	142

Symmetry codes: (i) 
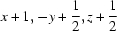
; (ii) 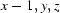; (iii) 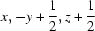; (iv) 
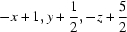
; (v) 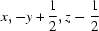.

**Fig. 1.**
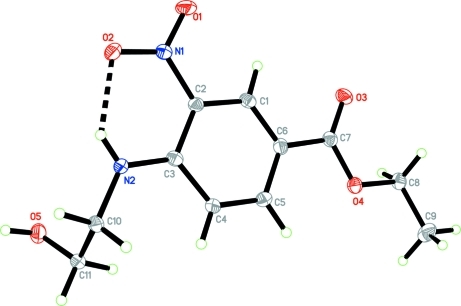
The molecular structure of the title compound, showing 50% probability displacement ellipsoids and the atom-numbering scheme. Hydrogen atoms are shown as spheres of arbitrary radius. The dashed line indicates an intramolecular hydrogen bond.

**Fig. 2.**
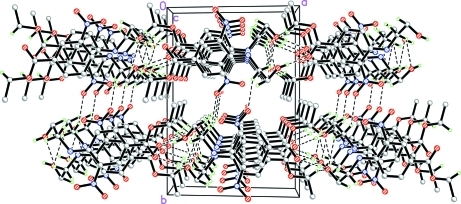
The crystal packing of the title compound, viewed along the c axis. H atoms not involved in the intermolecular interactions (dashed lines) have been omitted for clarity.

## supplementary crystallographic information

### Comment

Benzimidazoles serve as a common scaffold used worldwide for various successful drugs (Mayer *et al.*, 1998). Construction of pharmacologically important benzimidazoles could be accessed *via* nitrobenzoic acid precursors (Brouillette *et al.*, 1999; Williams *et al.*, 1995; Wright, 1951). The title compound was obtained as an intermediate in the synthesis of benzimidazole derivatives; we present here its crystal structure.

In the title compound (Fig. 1), the molecular structure is stabilized by an intramolecular N2—H2A···O2 hydrogen bond which generates an *S*(6) ring motif (Bernstein *et al.*, 1995). The nitro group is slightly twisted away from the benzene ring, the dihedral angle between N1/O1/O2/C2 and C1–C6 being 5.2 (2)°. The bond lengths (Allen *et al.*, 1987) and angles in the molecule are within normal ranges and are similiar to those in other related structures (Narendra Babu, Abdul Rahim, Abd Hamid *et al.*, 2009; Narendra Babu, Abdul Rahim, Osman *et al.*, 2009).

In the crystal packing (Fig. 2), intermolecular O5—H5B···O3, C8—H8A···O5, C10—H10A···O5, C10—H10B···O1 and C11—H11A···O2 hydrogen bonds (Table 1) link the molecules into a three-dimensional network.

### Experimental

The synthesis of the title compound was performed by the dropwise addition of *N*,*N*-diisopropyl ethylamine (1.1 mmol) to a stirred solution of ethyl 4-fluoro-3-nitrobenzoate (1.0 mmol) in dry dichloromethane (10.0 ml), followed by ethanolamine (1.1 mmol). The reaction mixture was left stirring overnight at room temperature under an inert atmosphere. Upon completion, the reaction mixture was washed with 10% Na_2_CO_3_ (3 x 10.0 ml). The combined organic fractions were dried over MgSO_4_ and evaporated *in vacuo*. Recrystallisation with hot hexane gave the title compound as bright yellow crystals, which were found to be suitable for characterisation by X-ray crystallography.

### Refinement

H2A and H5B were located in a difference Fourier map and were refined freely [N—H = 0.84 (3) Å; O—H = 0.83 (3) Å]. The remaining H atoms were positioned geometrically [C—H = 0.93 to 0.97 Å] and were refined using a riding model, with *U*_iso_(H) = x*U*_eq_(C), where x = 1.5 for methyl H and 1.2 for all other H atoms. A rotating group model was applied to the methyl group. The crystal studied was a non-merohedral twin, the refined ratio of the twin components being 0.264 (2):0.736 (2).

### Crystal data

**Table:**

C_11_H_14_N_2_O_5_	*F*(000) = 536
*M**_r_* = 254.24	*D*_x_ = 1.491 Mg m^−^^3^
Monoclinic, *P*2_1_/*c*	Mo *K*α radiation, λ = 0.71073 Å
Hall symbol: -P 2ybc	Cell parameters from 1880 reflections
*a* = 10.6422 (6) Å	θ = 2.4–28.1°
*b* = 14.9954 (9) Å	µ = 0.12 mm^−^^1^
*c* = 7.1975 (4) Å	*T* = 100 K
β = 99.607 (2)°	Needle, yellow
*V* = 1132.50 (11) Å^3^	0.43 × 0.13 × 0.03 mm
*Z* = 4	

### Data collection

**Table:**

Bruker SMART APEXII CCD area-detector diffractometer	2587 independent reflections
Radiation source: fine-focus sealed tube	2026 reflections with *I* > 2σ(*I*)
graphite	*R*_int_ = 0.050
φ and ω scans	θ_max_ = 27.5°, θ_min_ = 2.4°
Absorption correction: multi-scan (*SADABS*; Bruker, 2090)	*h* = −13→13
*T*_min_ = 0.951, *T*_max_ = 0.997	*k* = −19→19
8457 measured reflections	*l* = −8→9

### Refinement

**Table:**

Refinement on *F*^2^	Primary atom site location: structure-invariant direct methods
Least-squares matrix: full	Secondary atom site location: difference Fourier map
*R*[*F*^2^ > 2σ(*F*^2^)] = 0.051	Hydrogen site location: inferred from neighbouring sites
*wR*(*F*^2^) = 0.129	H atoms treated by a mixture of independent and constrained refinement
*S* = 1.04	*w* = 1/[σ^2^(*F*_o_^2^) + (0.0602*P*)^2^ + 0.3204*P*] where *P* = (*F*_o_^2^ + 2*F*_c_^2^)/3
2587 reflections	(Δ/σ)_max_ < 0.001
173 parameters	Δρ_max_ = 0.50 e Å^−^^3^
0 restraints	Δρ_min_ = −0.31 e Å^−^^3^

### Special details

**Table:**

Experimental. The crystal was placed in the cold stream of an Oxford Cryosystems Cobra open-flow nitrogen cryostat (Cosier & Glazer, 1986) operating at 100.0 (1) K.
Geometry. All esds (except the esd in the dihedral angle between two l.s. planes) are estimated using the full covariance matrix. The cell esds are taken into account individually in the estimation of esds in distances, angles and torsion angles; correlations between esds in cell parameters are only used when they are defined by crystal symmetry. An approximate (isotropic) treatment of cell esds is used for estimating esds involving l.s. planes.
Refinement. Refinement of *F*^2^ against ALL reflections. The weighted *R*-factor wR and goodness of fit *S* are based on *F*^2^, conventional *R*-factors *R* are based on *F*, with *F* set to zero for negative *F*^2^. The threshold expression of *F*^2^ > σ(*F*^2^) is used only for calculating *R*-factors(gt) etc. and is not relevant to the choice of reflections for refinement. *R*-factors based on *F*^2^ are statistically about twice as large as those based on *F*, and *R*- factors based on ALL data will be even larger.

### Fractional atomic coordinates and isotropic or equivalent isotropic displacement parameters (Å^2^)

**Table:**

	*x*	*y*	*z*	*U*_iso_*/*U*_eq_	
O1	0.38669 (15)	0.04338 (11)	1.1300 (3)	0.0285 (4)	
O2	0.57298 (13)	0.09227 (10)	1.2508 (2)	0.0193 (4)	
O3	0.04479 (14)	0.22764 (10)	0.8325 (2)	0.0194 (4)	
O4	0.07343 (13)	0.37665 (10)	0.8413 (2)	0.0170 (4)	
O5	0.82105 (15)	0.33071 (11)	1.0883 (2)	0.0192 (4)	
N1	0.46108 (17)	0.10564 (12)	1.1729 (3)	0.0157 (4)	
N2	0.61527 (17)	0.26565 (12)	1.2825 (3)	0.0147 (4)	
C1	0.29266 (19)	0.20462 (14)	1.0362 (3)	0.0141 (5)	
H1A	0.2445	0.1539	0.9998	0.017*	
C2	0.4166 (2)	0.19539 (14)	1.1327 (3)	0.0135 (4)	
C3	0.49466 (19)	0.27115 (14)	1.1919 (3)	0.0135 (4)	
C4	0.43542 (19)	0.35571 (14)	1.1508 (3)	0.0148 (5)	
H4A	0.4811	0.4071	1.1901	0.018*	
C5	0.3135 (2)	0.36374 (14)	1.0555 (3)	0.0148 (5)	
H5A	0.2784	0.4202	1.0308	0.018*	
C6	0.2402 (2)	0.28743 (14)	0.9938 (3)	0.0150 (5)	
C7	0.11002 (19)	0.29283 (14)	0.8828 (3)	0.0148 (5)	
C8	−0.05110 (19)	0.38757 (14)	0.7226 (3)	0.0168 (5)	
H8A	−0.1143	0.3517	0.7709	0.020*	
H8B	−0.0473	0.3685	0.5949	0.020*	
C9	−0.0866 (2)	0.48440 (16)	0.7242 (4)	0.0254 (6)	
H9A	−0.1695	0.4929	0.6501	0.038*	
H9B	−0.0251	0.5192	0.6722	0.038*	
H9C	−0.0879	0.5031	0.8515	0.038*	
C10	0.69687 (19)	0.34151 (14)	1.3465 (3)	0.0142 (4)	
H10A	0.7673	0.3210	1.4400	0.017*	
H10B	0.6484	0.3841	1.4073	0.017*	
C11	0.75012 (19)	0.38863 (14)	1.1886 (3)	0.0157 (5)	
H11A	0.6801	0.4141	1.1011	0.019*	
H11B	0.8047	0.4372	1.2419	0.019*	
H2A	0.642 (2)	0.2141 (18)	1.310 (4)	0.021 (7)*	
H5B	0.888 (3)	0.3148 (18)	1.156 (4)	0.028 (8)*	

### Atomic displacement parameters (Å^2^)

**Table:**

	*U*^11^	*U*^22^	*U*^33^	*U*^12^	*U*^13^	*U*^23^
O1	0.0224 (8)	0.0119 (8)	0.0480 (12)	−0.0046 (7)	−0.0035 (8)	0.0003 (8)
O2	0.0169 (8)	0.0143 (8)	0.0252 (9)	0.0027 (6)	−0.0004 (7)	0.0010 (7)
O3	0.0145 (7)	0.0177 (8)	0.0243 (9)	−0.0040 (6)	−0.0011 (7)	0.0005 (7)
O4	0.0118 (7)	0.0168 (8)	0.0207 (9)	0.0003 (6)	−0.0024 (6)	0.0005 (7)
O5	0.0149 (8)	0.0216 (9)	0.0204 (9)	0.0025 (6)	0.0012 (7)	−0.0020 (7)
N1	0.0146 (9)	0.0129 (9)	0.0192 (10)	−0.0015 (7)	0.0018 (7)	−0.0009 (8)
N2	0.0116 (8)	0.0115 (9)	0.0201 (10)	0.0007 (7)	0.0002 (8)	0.0017 (8)
C1	0.0153 (11)	0.0145 (10)	0.0130 (11)	−0.0038 (8)	0.0036 (8)	−0.0026 (9)
C2	0.0151 (10)	0.0119 (10)	0.0144 (11)	−0.0002 (8)	0.0051 (8)	0.0017 (8)
C3	0.0145 (10)	0.0142 (11)	0.0118 (11)	−0.0018 (8)	0.0029 (8)	−0.0001 (8)
C4	0.0135 (10)	0.0123 (10)	0.0188 (12)	−0.0012 (8)	0.0030 (8)	−0.0016 (9)
C5	0.0169 (10)	0.0128 (10)	0.0147 (11)	0.0014 (8)	0.0027 (8)	0.0010 (9)
C6	0.0132 (10)	0.0165 (11)	0.0156 (11)	0.0003 (8)	0.0030 (9)	0.0013 (9)
C7	0.0148 (10)	0.0159 (11)	0.0144 (11)	−0.0004 (8)	0.0045 (9)	0.0007 (9)
C8	0.0116 (10)	0.0203 (11)	0.0161 (11)	0.0004 (8)	−0.0045 (9)	0.0015 (9)
C9	0.0198 (11)	0.0226 (13)	0.0301 (14)	0.0053 (9)	−0.0069 (10)	−0.0019 (11)
C10	0.0116 (9)	0.0147 (10)	0.0148 (11)	−0.0009 (8)	−0.0017 (8)	−0.0004 (9)
C11	0.0127 (10)	0.0128 (10)	0.0208 (11)	−0.0013 (8)	0.0005 (9)	−0.0004 (9)

### Geometric parameters (Å, °)

**Table:**

O1—N1	1.230 (2)	C4—C5	1.368 (3)
O2—N1	1.245 (2)	C4—H4A	0.9300
O3—C7	1.218 (3)	C5—C6	1.414 (3)
O4—C7	1.335 (3)	C5—H5A	0.9300
O4—C8	1.461 (2)	C6—C7	1.482 (3)
O5—C11	1.424 (3)	C8—C9	1.501 (3)
O5—H5B	0.83 (3)	C8—H8A	0.9700
N1—C2	1.440 (3)	C8—H8B	0.9700
N2—C3	1.342 (3)	C9—H9A	0.9600
N2—C10	1.459 (3)	C9—H9B	0.9600
N2—H2A	0.84 (3)	C9—H9C	0.9600
C1—C6	1.375 (3)	C10—C11	1.526 (3)
C1—C2	1.391 (3)	C10—H10A	0.9700
C1—H1A	0.9300	C10—H10B	0.9700
C2—C3	1.430 (3)	C11—H11A	0.9700
C3—C4	1.425 (3)	C11—H11B	0.9700
			
C7—O4—C8	116.01 (16)	O3—C7—C6	123.46 (19)
C11—O5—H5B	110 (2)	O4—C7—C6	112.56 (18)
O1—N1—O2	121.21 (17)	O4—C8—C9	107.99 (17)
O1—N1—C2	118.86 (17)	O4—C8—H8A	110.1
O2—N1—C2	119.92 (17)	C9—C8—H8A	110.1
C3—N2—C10	125.21 (19)	O4—C8—H8B	110.1
C3—N2—H2A	115.5 (18)	C9—C8—H8B	110.1
C10—N2—H2A	119.1 (18)	H8A—C8—H8B	108.4
C6—C1—C2	121.12 (19)	C8—C9—H9A	109.5
C6—C1—H1A	119.4	C8—C9—H9B	109.5
C2—C1—H1A	119.4	H9A—C9—H9B	109.5
C1—C2—C3	121.68 (19)	C8—C9—H9C	109.5
C1—C2—N1	116.49 (18)	H9A—C9—H9C	109.5
C3—C2—N1	121.82 (19)	H9B—C9—H9C	109.5
N2—C3—C4	120.65 (19)	N2—C10—C11	113.65 (18)
N2—C3—C2	123.9 (2)	N2—C10—H10A	108.8
C4—C3—C2	115.47 (18)	C11—C10—H10A	108.8
C5—C4—C3	122.1 (2)	N2—C10—H10B	108.8
C5—C4—H4A	118.9	C11—C10—H10B	108.8
C3—C4—H4A	118.9	H10A—C10—H10B	107.7
C4—C5—C6	120.9 (2)	O5—C11—C10	112.94 (18)
C4—C5—H5A	119.5	O5—C11—H11A	109.0
C6—C5—H5A	119.5	C10—C11—H11A	109.0
C1—C6—C5	118.61 (19)	O5—C11—H11B	109.0
C1—C6—C7	118.53 (19)	C10—C11—H11B	109.0
C5—C6—C7	122.85 (19)	H11A—C11—H11B	107.8
O3—C7—O4	123.96 (19)		
			
C6—C1—C2—C3	0.0 (3)	C3—C4—C5—C6	0.3 (3)
C6—C1—C2—N1	178.9 (2)	C2—C1—C6—C5	−1.9 (3)
O1—N1—C2—C1	−3.9 (3)	C2—C1—C6—C7	177.1 (2)
O2—N1—C2—C1	176.5 (2)	C4—C5—C6—C1	1.8 (3)
O1—N1—C2—C3	175.0 (2)	C4—C5—C6—C7	−177.2 (2)
O2—N1—C2—C3	−4.6 (3)	C8—O4—C7—O3	−2.2 (3)
C10—N2—C3—C4	−0.1 (3)	C8—O4—C7—C6	176.60 (18)
C10—N2—C3—C2	−179.3 (2)	C1—C6—C7—O3	2.8 (3)
C1—C2—C3—N2	−178.8 (2)	C5—C6—C7—O3	−178.3 (2)
N1—C2—C3—N2	2.4 (3)	C1—C6—C7—O4	−176.1 (2)
C1—C2—C3—C4	2.0 (3)	C5—C6—C7—O4	2.9 (3)
N1—C2—C3—C4	−176.81 (19)	C7—O4—C8—C9	168.7 (2)
N2—C3—C4—C5	178.7 (2)	C3—N2—C10—C11	−76.3 (3)
C2—C3—C4—C5	−2.1 (3)	N2—C10—C11—O5	−57.4 (2)

### Hydrogen-bond geometry (Å, °)

**Table:**

*D*—H···*A*	*D*—H	H···*A*	*D*···*A*	*D*—H···*A*
N2—H2A···O2	0.84 (3)	1.99 (3)	2.642 (2)	134 (2)
O5—H5B···O3^i^	0.83 (3)	2.02 (3)	2.851 (2)	177 (3)
C8—H8A···O5^ii^	0.97	2.51	3.271 (3)	135
C10—H10A···O5^iii^	0.97	2.54	3.267 (3)	132
C10—H10B···O1^iv^	0.97	2.43	3.168 (3)	133
C11—H11A···O2^v^	0.97	2.59	3.403 (3)	142

Symmetry codes: (i) *x*+1, −*y*+1/2, *z*+1/2; (ii) *x*−1, *y*, *z*; (iii) *x*, −*y*+1/2, *z*+1/2; (iv) −*x*+1, *y*+1/2, −*z*+5/2; (v) *x*, −*y*+1/2, *z*−1/2.
